# From Early Models to Emerging Trends: The Evolution of Computational Hemolysis Prediction

**DOI:** 10.1007/s10439-026-04063-3

**Published:** 2026-03-19

**Authors:** Ilaria Guidetti, Maria Laura Costantino, Francesco De Gaetano

**Affiliations:** Politecnico di Milano, Department of Chemistry, Materials and Chemical Engineering “Giulio Natta”, LaBS, Piazza Leonardo da Vinci, 32, 20133 Milan, Italy

**Keywords:** Mechanical blood damage, Hemolysis prediction models, Stress-based models, Strain-based models, Lagrangian approach, Eulerian approach

## Abstract

Mechanical hemolysis remains one of the most critical complications associated with blood-contacting devices. Computational prediction of blood damage has been widely adopted as a key tool for evaluating the hemolytic potential of cardiovascular devices. Although numerical hemolysis modeling has been investigated since the 1990s, a universally accurate and predictive approach is still lacking. This review traces the evolution of computational hemolysis models, from the earliest stress-based power-law formulation to recent strain-based approaches that describe the red blood cells at cellular and molecular levels. It examines the various definitions of the power-law shear stress found in literature, including Von Mises-like formulations, extensions incorporating extensional or Reynolds stresses and those based on turbulent dissipation rate or deformation of the red blood cell. The broad range of power-law constants reported in literature is summarized, together with their development conditions. Furthermore, the Lagrangian and Eulerian approaches used for numerical hemolysis predictions are analyzed in detail. Finally, emerging trends and future directions are highlighted, offering insights into the pathways toward more reliable hemolysis modeling.

**Supplementary Information:**

The online version contains supplementary material available at 10.1007/s10439-026-04063-3.

## Introduction

Mechanical hemolysis consists of the rupture or damage of the red blood cell (RBC) membrane, called stroma, due to prolonged exposure to non-physiological high shear stresses and consequent dispersion of the intracellular hemoglobin in plasma. In the body, erythrocytes are subject to shear rates up to 1600 s^−1^ and shear stresses between 0.1 and 15 Pa; instead, when passing through cardiovascular devices such as blood pumps, shear rates are generally above 10000 s^−1^ and stresses can reach up to 200 Pa [1–3]. This results in the RBCs exposure to non-physiological high shear stresses for prolonged durations, increasing the risk for hemolysis.

The phenomenon of mechanical hemolysis has been observed since the first implants of cardiac valve prostheses in the 1950s and 1960s [4]. Over the years, the percentage of hemolysis generated by cardiovascular devices has decreased thanks to a better knowledge of blood fluid dynamics, better designs, the introduction of more biocompatible materials and the development of new processing technologies, such as computer numerical control, micromachinery, and laser machinery [5].

Nevertheless, clinical and subclinical hemolytic anemia (HA) is still observable and its treatment is of critical importance. Clinical HA can be identified when patients with cardiovascular devices suffer from anemia, present an acceleration of the RBCs production and signs of their lysis. If anemia is not found but the other two factors are present, then it is referred to as subclinical HA [4]. This disorder has been associated with various clinical complications affecting multiple organ systems, including the gastrointestinal, cardiovascular, pulmonary, urogenital, and renal systems [6].

Modern devices strongly reduced HA incidence; however, subclinical HA still occurs in up to 51% of patients with mechanical aortic valve replacements. Moreover, hemolysis remains a recognized complication of mechanical circulatory support devices and of cardiopulmonary bypass, extracorporeal membrane oxygenation, and hemodialysis circuits with incidences up to 18% in both ventricular assist devices and extracorporeal membrane oxygenation circuits [7–12].

Given its clinical implications, hemolysis assessment is a critical factor in the regulatory approval process for new cardiovascular devices. Extensive research efforts are dedicated to evaluating hemolysis risk, with the ultimate goal of minimizing its occurrence and improving patient outcomes.

Computational modeling has become a widely adopted approach for evaluating mechanical hemolysis induced by cardiovascular devices. It typically integrates a numerical hemolysis model with computational fluid dynamics (CFD) simulations of the device under investigation. Once experimentally validated, numerical models offer a cost-effective and time-efficient tool that can significantly reduce the number of in vitro and in vivo testing. By relying primarily on computational processing time rather than complex laboratory procedures, they also enable better planning of in vivo studies and help mitigate associated experimental risks.

Numerical models play a crucial role in the design and optimization of cardiovascular devices, enabling a preliminary assessment of hemolysis risk. These models typically involve the solution of the pressure and velocity fields within the device, from which secondary hemodynamic quantities are derived and subsequently used to estimate blood damage. Unlike in vitro tests, which provide an overall blood damage evaluation of hemolysis, numerical methods can pinpoint regions of high shear stress or turbulent flow, thereby guiding targeted design modifications and allowing improvement in performance [13].

Over the years, these approaches have been extensively explored and refined, resulting in increasingly sophisticated computational hemolysis models. Nevertheless, the appropriate methodology for deriving hemolysis from computed pressure and velocity distributions remains an open challenge. As a result, a universally accepted standard for blood damage evaluation remains elusive and no existing model can yet predict hemolysis accurately across a broad range of medical devices.

This review provides a comprehensive overview of the numerical models and approaches developed for hemolysis prediction, tracing their evolution from the earliest formulations to the most recent advances reported in the literature. It begins with a brief introduction to red blood cells and their key characteristics relevant to hemolysis modeling. Next, it discusses current methodologies used to evaluate hemolysis in vitro and highlights the ongoing challenges in accurately assessing blood damage. Finally, the review examines the broad spectrum of computational models for hemolysis prediction, addressing longstanding debates and emerging trends in the field.

## Red Blood Cell Characteristics

Red blood cells have a biconcave disk shape that gives them 44% more surface area for gas exchange than a sphere of similar volume [14]. This shape also contributes to the high deformability of these cells, which are able to pass through capillaries much smaller than their diameter, which is about 8 μm.

The high deformability of these cells is also due to the lack of nucleus and to the spectrin network that constitutes their cellular membrane. The latter provides great elasticity to this viscoelastic membrane, while the viscous component is represented by a bilayer of phospholipids [5].

The RBC membrane can undergo poration due to strain and it can withstand up to 6% deformation before starting to rupture [15]. Both the rupture and the increase of the pore sizes above the diameter of hemoglobin (about 5 nm) lead to the release of this protein into plasma. In physiological conditions, the total concentration of hemoglobin present in blood is 12.0–13.5 g/dL and only 0.008 g/dL is present outside of RBCs [5]. Plasma-free hemoglobin (pfHb) concentrations above 0.03–0.05 g/dL can cause several complications, including hemolytic anemia, renal impairment, and multiple organ failure [5]. In particular, excess pfHb reduces the availability of nitric oxide in blood, which plays a key role in platelet activation, smooth muscle tone regulation, and vascular homeostasis [6].

RBCs arrangement and interaction with plasma depend on the flow characteristics and the variations that occur cause blood to be a non-Newtonian fluid with shear-thinning and viscoelastic properties. At null or low shear rates ($\dot{\gamma }$ < 20–40 s^−1^), blood has a high apparent viscosity due to the RBCs aggregation in coin-stack-like microstructures called rouleaux [16]. These gradually disaggregate with the increase of the shear rate until only single cells are left, each with a specific relaxation time (increasing from 100 to 400 ms as the cell ages) [17]. These cells maintain the biconcave shape and present a tumbling motion: the whole cell rotates inside the flow [5]. An additional increase in shear rate leads RBCs to deform into an ellipsoidal shape and align their orientation according to the flow direction, causing a decrease in blood viscosity. In this flow condition, RBCs are characterized by a tank-treading motion: the cellular membrane rotates around the intracellular fluid while the cell maintains a constant inclination angle relative to the flow. This type of motion is attributed to the RBC’s favorable surface-area-to-volume ratio, which enables significant deformation without an increase in membrane area. This behavior is maintained until the shear rate increase induces further elongation, membrane poration, and ultimately its rupture [18].

## Experimental Evaluation of Hemolysis in Cardiovascular Devices

Experimental hemolysis evaluation is a regulatory requirement for the approval and clinical use of blood-contacting devices. In particular, the International Organization for Standardization (ISO) defined in the UNI EN ISO 10993-4:2017 the requirements and classification of blood-contacting devices, together with principles for in vitro evaluation of the hemolytic risk. Moreover, different protocols have been developed for specific devices, such as the ASTM F1841-19^ε1^ standard which provides instructions for assessing hemolysis in continuous blood pumps.

The experimental evaluation of the blood damage risk is performed through the measurement of the increase in pfHb over a prolonged use of the device. The measuring technique suggested by the ASTM F756-17 is the cyanmethemoglobin detection method which uses a spectrophotometer. This and other pfHb measurement techniques were evaluated by Fairbanks et al. and Malinauskas et al., who reported that most methods exhibit good accuracy in the absence of interfering substances [19, 20]. Moreover, direct optical approaches, such as the Cripps method, were shown to represent a reliable and accurate option and, in some cases, outperform other techniques even in the presence of interferents.

Currently, experimental hemolysis results are often reported using the modified index of hemolysis (MIH), which was proposed in 1993 by Mueller et al. with the aim of standardizing blood damage reported data across different test conditions and research groups [21]. Equation 1 shows this index as indicated in the ASTM F1841-19^ε1^ standard:

1 $${\mathrm{MIH}}\left[ - \right] = \frac{{\Delta {\mathrm{pfHb}} \cdot V \cdot \frac{100 - Ht}{{100}}}}{{Q \cdot \Delta T \cdot {\mathrm{Hb}}}}$$

In Eq. 1, $\Delta{\mathrm{pfHb}}$ (mg/dL) is the change in plasma free hemoglobin concentration over time, $V$ (mL) is the total blood volume used in the test, $\mathrm{Ht}$ (%) is the blood hematocrit, $Q$ (L/min) is the flow rate, $\Delta T$ (min) is the sampling interval during the experiment, and $\mathrm{Hb}$ (g/dL) is the total hemoglobin concentration of blood.

Despite the standardization efforts, interpreting and comparing hemolysis test results remain challenging. In fact, intra- and inter-laboratory reproducibility of hemolysis experimental tests is often low, as highlighted by Herbertson et al. and von Petersdorff-Campen and Daners [22, 23]. In the first study, the intra-laboratory variability reached up to 94%, while the inter-laboratory variability up to 85%. In the second paper, they examined hemolysis studies on VADs from 2020 and found intra-laboratory variations up to 51%. The factors that contribute to this low reproducibility are related to characteristics, sourcing, and handling of blood and have been extensively studied in literature; however, a comprehensive understanding of their effects is lacking and there are insufficient measures to be implemented during testing to diminish the variability.

Conducting hemolysis tests involves a high workload due to their long duration, high number of samples to collect and analyze, control and regulation of the setup and, in some studies, the need to conduct multiple tests in parallel or to perform multiple repetitions. Many of these aspects are related to the low precision of the pfHb concentration measurements and the difficulty to detect low variations.

Blood sourcing is one of the most significant challenges related to blood damage evaluation; in fact, these tests can require high blood volumes and have strict requirements on the timing between collection and use. The ideal condition would involve the use of fresh human blood; however, this is not possible for many laboratories and for tests requiring large amounts of blood. Abattoirs are one of the sources used to overcome these issues, but blood could be damaged before starting the experiment due to uncontrolled collection procedures. Heterogeneity in the sources used contributes to further reducing reproducibility, especially when different species are used as donors, given the differences in their blood properties [24].

Recently, Ponnaluri et al. demonstrated that hemolysis test reproducibility can be substantially improved, reporting coefficients of variation below 16% for pfHb measurements. This result was achieved through careful selection of blood donors, an optimized study design, and the use of small blood volumes. While their approach is not directly applicable to large-scale devices, it provides valuable insights into the influence of blood species and hematocrit on hemolysis measurements and highlights potential strategies for reducing experimental variability [25].

Due to these challenges, the evaluation of the hemolytic potential of new devices for regulatory purposes can only be performed through comparisons with similar devices already in clinical use and whose safety has been proven. Several strategies are being explored to address these challenges and enhance the reliability and efficiency of hemolysis assessment. One approach involves the use of fluids that mimic the shear sensitivity of blood while being easier to standardize and analyze [26–28]. However, replicating the complex, time-dependent mechanical behavior of red blood cells remains a significant limitation. Another promising strategy is the implementation of continuous blood damage monitoring, which replaces manual sampling and analysis with automated, real-time assessment. While this could reduce experimental effort, duration, and measurement errors, current technologies may still lack the necessary sensitivity and accuracy [23]. Other approaches focus on minimizing the blood volume required for testing, with particular emphasis on miniaturizing the test loop used to evaluate hemolysis in rotary blood pumps [29]. Overcoming the challenges related to hemolysis in vitro evaluation is crucial not only for improving reliability but also for streamlining the development of cardiovascular devices. Enhancing the efficiency of this validation process can accelerate innovation, reduce costs, and testing times and ultimately lead to faster and more effective treatments for patients.

## Experimental Foundations of Hemolysis Modeling

Most current numerical hemolysis models are derived from the power-law equation proposed in 1990 by Giersiepen et al. [30]. This model was derived from experimental data obtained by Wurzinger et al. using an axial Couette viscometer designed by Heuser and Opitz and able to expose blood to uniform shear for short time periods [31, 32]. The amount of pfHb released as a function of shear stress and exposure time was approximated using a two-dimensional curve fitting technique and used to determine the parameters $A$, $B,$ and $C$ for the power-law model by regression analysis. The power-law formulation is presented in Eq. 2:

2 $${\mathrm{HI}}\left( \% \right) = \frac{{{\mathrm{pfHb}}}}{{{\mathrm{Hb}}}}*100 = C*t_{\exp }^{A} *\tau^{B}$$

where $HI\left(\%\right)$ is the hemolysis index, and ${t}_{exp}$ (s) is the exposure time of blood to the shear stress $\tau$ (N/m^2^).

**Fig. 1:**
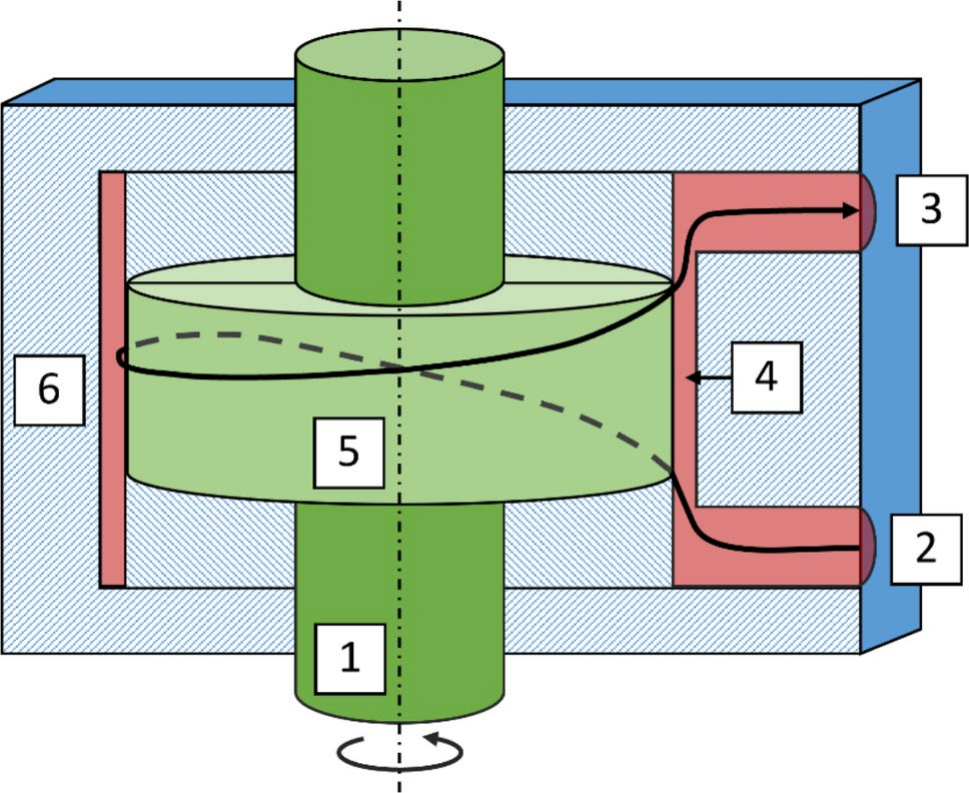
Schematic axial Couette viscometer by Heuser and Opitz: (1) driving shaft; (2) inlet; (3) outlet; (4) shearing gap; (5) inner rotating cylinder; (6) outer stationary cylinder. A blood particle follows the pathway described by the bold arrow

Figure 1 shows a schematic representation of the shearing device used by both Wurzinger et al. and Heuser and Opitz, whose parameter set has been reported in detail for the first time by Song et al. in 2003 [32, 33]. As noted by Faghih and Sharp in 2019, there has been confusion in the literature regarding the value of parameter $C$ defined from Heuser and Opitz’s experiments [5]. In particular, the uncertainty arose from an inconsistent use of the parameter $C$ value with respect to the formulation used for the power-law, either employed as a unit or as a percentage hemolysis index. Several studies erroneously used 1.8000*10^−6^, even though they defined the hemolysis index in percentage, while a comparison with figures 11 and 12 in the original paper shows that the correct value for parameter $C$ should be 1.8000*10^−4^ when used to compute $HI(\%)$ [34–37].

The shearing device used in these first experiments has been shown to overestimate hemolysis, likely due to secondary blood damage caused by friction and heat near the mechanical seal. For this reason, Giersiepen’s parameters are often found to significantly overpredict hemolysis.

**Fig. 2:**
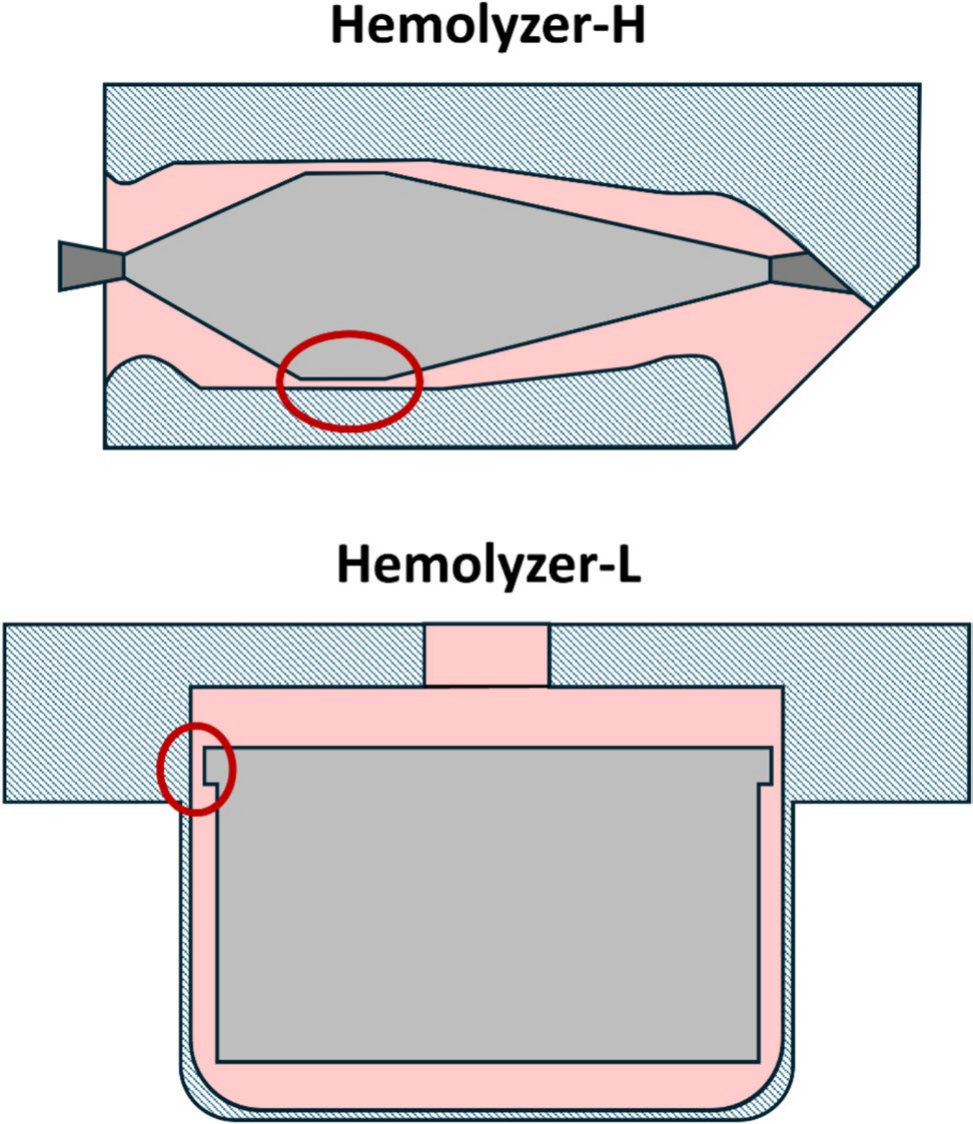
Schematics of Hemolyzer-H and Hemolyzer-L with the gaps highlighted

Since 2011, new types of Couette viscometers have been developed and widely used to derive parameter sets for the power-law model. Zhang et al. designed two seal-free Couette devices, adapted from centrifugal blood pumps [38]. The Hemolyzer-H is an axial-flow Couette device, created by replacing the rotor of the Jarvik 2000® pump with a titanium spindle. In contrast, the Hemolyzer-L is derived from the CentriMag® blood pump and produces a centrifugal flow. In both devices, blood damage is primarily induced in the gap region, with negligible damage elsewhere. A schematic description of the two viscometers is shown in Figure 2.

The same hemolyzers were subsequently used by Ding et al. to determine the parameter sets for various species: ovine, bovine, porcine and human [39].

The experimental data collected by Zhang et al. were also used by Gesenhues et al. to derive a set of parameters tailored to a power-law model in which the shear stress is substituted with an effective stress [40]. The latter is computed from the RBC deformation through the strain-based model developed by Arora et al., which will be discussed in “Strain-based Hemolysis Models” section [41]. Gesenhues et al. observed that when Zhang’s parameters were used with the strain-based model, the predicted hemolysis was significantly underreported. Using standard nonlinear regression techniques, they derived a new set of parameters that more accurately matched the experimental results when applied in combination with Arora’s model.

Despite the widespread use of Couette viscometers to evaluate blood damage and derive power-law parameters, they are not the only devices that have been used for this purpose. Other approaches are described in Online Appendix A and are not reported in Table 1 due to their limited adoption in the literature and the presence of potential sources of error in their experimental setups.

**Table 1:** Summary of parameter sets available in literature for the power-law ($HI\left(\%\right)=C*{t}_{exp}^{A}*{\uptau }^{B}$), derived from experiments using Couette viscometers

Source	C	A	B	Species	Test conditions
Giersiepen 1990 [30]	3.6200*10^−5^	0.7850	2.4160	Human RBC	$\tau$: 57-255 Pa ${t}_{exp}$:7-700 ms
Heuser 1980 [32]	1.8000*10^−4^	0.7650	1.9910	Porcine	$\tau$: 40-700 Pa ${t}_{exp}$:3-690 ms
Zhang 2011 [38]	1.2280*10^−5^	0.6606	1.9918	Ovine	$\tau$: 30-320 Pa ${t}_{exp}$:30-1500 ms
Ding 2015 [39]	6.7010*10^−4^	0.2778	1.0981	Porcine	$\tau$: 25-320 Pa ${t}_{exp}$:40-1500 ms
Ding 2015 [39]	3.4580*10^−6^	0.2777	2.0639	Human	$\tau$: 25-320 Pa ${t}_{exp}$:40-1500 ms
Ding 2015 [39]	9.7720*10^−5^	0.2076	1.4445	Bovine	$\tau$: 25-320 Pa ${t}_{exp}$:40-1500 ms
Gesenhues 2016 [40]	2.3212*10^−4^	0.3300	1.4924	Ovine	$\tau$: 30-320 Pa ${t}_{exp}$:30-1500 ms

## Computational Models for Hemolysis Prediction

Computational hemolysis prediction approaches based on the power-law can be referred to as continuum-based since they consider blood as a whole and do not model directly red blood cells and hemoglobin leakage. Other than the power-law, other continuum-based approaches have been developed for specific devices such as rotary blood pumps; however, they did not gain widespread adoption in the scientific community [42–45]. Since 2015, researchers have developed models at the cellular and molecular levels to predict hemolysis [46]. These approaches employ flow quantities computed through CFD to inform RBC simulations at the unit level, attempting to capture membrane deformation, pore formation, and hemoglobin leakage. By incorporating the physical and mechanical properties of the cell membrane, including membrane failure mechanics, these models can offer improved accuracy [46]. However, their high computational cost limits their applicability to full-scale cardiovascular devices [47, 48].

Numerical hemolysis models based on the power-law are typically classified according to their computational approach and the variables used to estimate blood damage. The first classification distinguishes between Eulerian and Lagrangian models: the former evaluates blood damage across the entire fluid domain within the cardiovascular device, while the latter assesses it along pathlines or trajectories extracted from the flow. The second classification categorizes models as stress-based, energy dissipation-based, or strain-based, depending on how shear stress or membrane strain is derived from the simulated flow field [49]. Stress-based models, the earliest developed, compute the hemolysis index directly from shear stress, obtained via velocity gradients in numerical simulations. Energy dissipation-based models establish a correlation between hemolysis and the viscous dissipation of turbulent kinetic energy, deriving the power-law term from the turbulent dissipation rate. Strain-based models, in contrast, estimate shear stress from alternative variables related to red blood cell deformation, typically inferred from velocity gradients in the fluid flow.

**Fig. 3:**
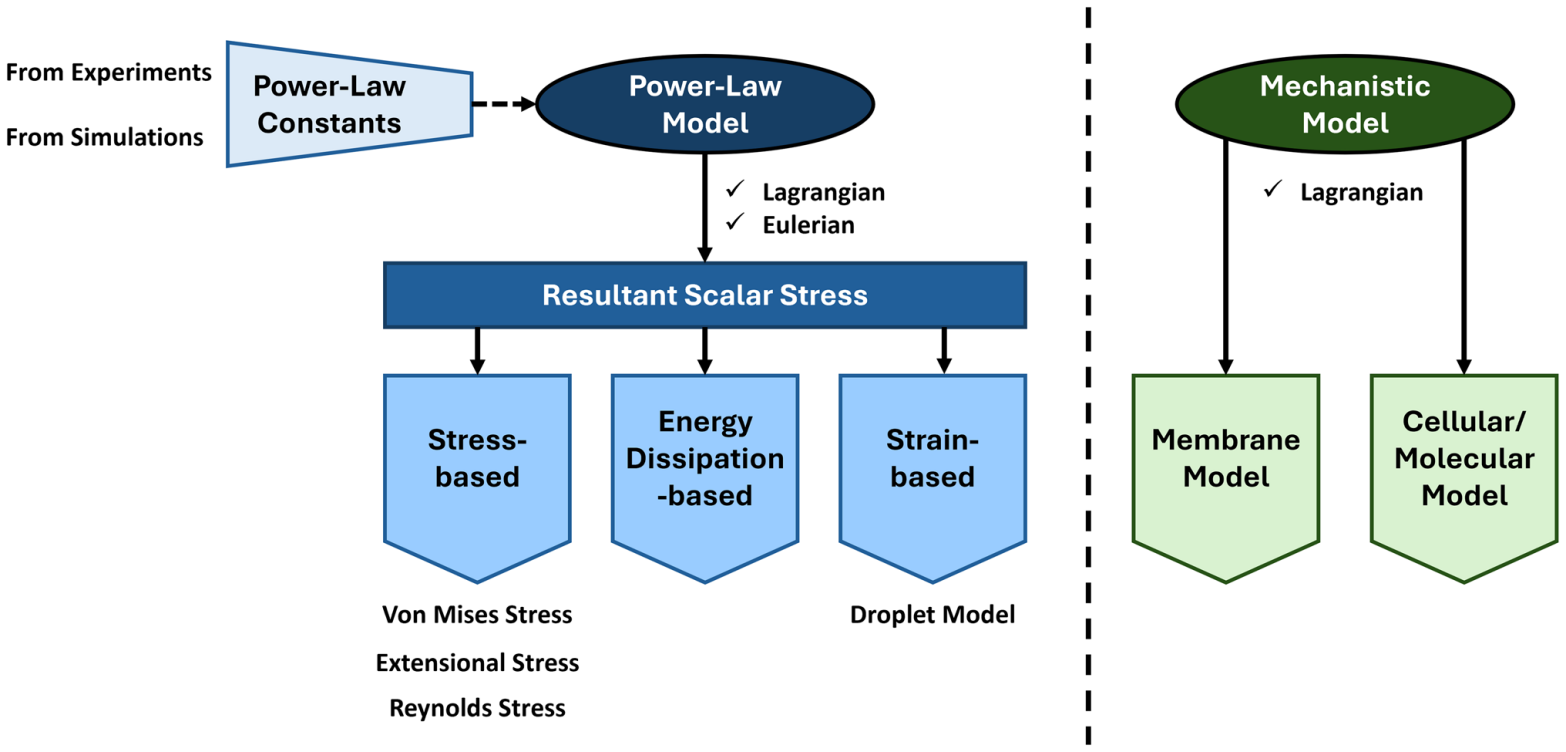
Schematic of the interconnections between models, topics and approaches employed for computational hemolysis prediction

Figure 3 presents a schematic overview of the main models, topics and approaches that have emerged throughout the development of computational hemolysis modeling, highlighting the interconnections between them. “Lagrangian or Eulerian Approach” section provides a detailed description of the two frameworks used to implement numerical hemolysis models. The following sections first discuss power-law models, tracing their evolution from stress-based to energy dissipation-based and finally to strain-based formulations (“Stress-based Hemolysis Models”, “Energy Dissipation-based Hemolysis Models”, and “Strain-based Hemolysis Models” sections, respectively). “Strain-based Hemolysis Models” section also illustrates strain-based approaches based on RBC membrane modeling, as well as models that simulate RBC behavior at the cellular and molecular level. “Power-Law Constants” section reviews the power-law constants reported in the literature, while “Emerging Trends in Computational Hemolysis Prediction” section explores emerging trends in numerical hemolysis prediction.

## Lagrangian or Eulerian Approach

Numerical hemolysis modeling was first performed employing a Lagrangian approach, with its earliest application in 1994 to evaluate shear stress variations and blood damage accumulation along particle streamlines in a rotary blood pump [50]. Pathlines for Lagrangian hemolysis computations can be extracted from flow field simulations using different methods. The most common approaches involve either extracting streamlines during post-processing or seeding particles at the device inlet, tracking their trajectories during the simulation, and extracting pathlines afterward. The first approach is primarily used for steady-state simulations, where streamlines and pathlines coincide, while the second is necessary for transient simulations [51].

Regardless of the extraction method, key variables recorded along the pathlines include velocity gradients, time instants, and spatial coordinates at each point. These are then used in post-processing to predict hemolysis. Depending on the modeling approach, the variables derived from CFD contribute to the calculations of key elements for hemolysis predictions, such as shear stress, cell deformation, and membrane tension.

Lagrangian approaches are largely employed to implement strain-based models, especially those simulating the RBC membrane at the molecular and cellular level. However, they were first introduced to integrate the power-law equation along a given pathline and determine the total hemolysis by accounting for cumulative damage. Five distinct formulations have been proposed for this damage accumulation process (Equations 3–7) [36]:

3 $${\mathrm{HI}}1\left( \% \right) = \mathop \sum \limits_{i = 1}^{N} C*\tau \left( {t_{i} } \right)^{{\mathrm{B}}} *\Delta t_{i}^{{\mathrm{A}}}$$

4 $${\mathrm{HI}}2\left( \% \right) = \mathop \sum \limits_{i = 1}^{N} A*C*\tau \left( {t_{i} } \right)^{{\mathrm{B}}} *t_{i}^{{{\mathrm{A}} - 1}} *\Delta t_{i}$$

5 $${\mathrm{HI}}3\left( \% \right) = C*\left( {\mathop \sum \limits_{i = 1}^{N} \Delta t_{i} *\tau \left( {t_{i} } \right)^{{{\mathrm{B}}/{\mathrm{A}}}} } \right)^{{\mathrm{A}}}$$

6 $${\mathrm{HI}}4\left( \% \right) = \mathop \sum \limits_{i = 1}^{N} C*A*\left[ {\mathop \sum \limits_{j = 1}^{i} \tau \left( {t_{j} } \right)^{{{\mathrm{B}}/{\mathrm{A}}}} *\Delta t_{j} + {\mathrm{DD}}\left( {t_{0} } \right)} \right]^{{{\mathrm{A}} - 1}} *\tau \left( {t_{i} } \right)^{{{\mathrm{B}}/{\mathrm{A}}}} *\Delta t_{i} \;{\mathrm{with}}\;{\mathrm{DD}} = \Delta t_{i} *\tau \left( {t_{i} } \right)^{{{\mathrm{B}}/{\mathrm{A}}}}$$

7 $${\mathrm{HI}}5\left( \% \right)\left( {t_{i} + \Delta t_{i} } \right) = C*\left( {t_{{{\mathrm{eff}}}} + \Delta t_{i} } \right)^{{\mathrm{A}}} *\left( {\tau \left( {t_{i} + \Delta t_{i} } \right)} \right)^{{\mathrm{B}}} \;{\mathrm{with}}\;t_{{{\mathrm{eff}}}} = \left( {\frac{{{\mathrm{HI}}5\left( \% \right)\left( {t_{i} } \right)}}{{C*\left( {\tau \left( {t_{i} + \Delta t_{i} } \right)} \right)^{{\mathrm{B}}} }}} \right)^{{1/{\mathrm{A}}}}$$

In these equations, $N$ represents the total number of steps along the pathline and $i$ is used as reference for the step under consideration. $\Delta t$ is the time difference between two consecutive steps and corresponds to the exposure time of the power-law equation ${t}_{\mathrm{exp}}$ in Eq. 2. In Eq. 6, $\mathrm{DD}$ is the mechanical damage dose while in Eq. 7, ${t}_{eff}$ is the effective time.

All the formulations reported in Equations 3–7 remain in use today, despite the known inaccuracies of $\mathrm{HI}1$ and $\mathrm{HI}2$, described in detail in Online Appendix B [37, 51, 52]. Furthermore, studies indicate that models incorporating blood damage history often produce comparable results: $\mathrm{HI}4$ predictions closely align with those of $\mathrm{HI}3$ and $\mathrm{HI}5$, which generally coincide [36, 51, 52]. The equivalence between $\mathrm{HI}3$ and $\mathrm{HI}5$ has been demonstrated for the first time, according to authors’ knowledge, in Online Appendix B.

Lagrangian models have been widely applied in the past for hemolysis prediction, but currently, most recent studies focus on the Eulerian approach [51, 53–55]. Despite this shift, Lagrangian models are still commonly applied in continuum-based models that account for red blood cell deformation, as well as in cellular and molecular-level models. This is largely due to the complexity or infeasibility of implementing these models with an Eulerian approach. In fact, Lagrangian methods allow performing hemolysis evaluation in a post-processing phase, which is crucial for modeling more intricate processes like membrane poration and hemoglobin leakage [56, 57].

The transition from Lagrangian to Eulerian models stems primarily from a key limitation of the former: their inability to fully cover the fluid domain with pathlines, particularly in devices with complex geometries [49]. The lack of full coverage can compromise the accuracy of hemolysis predictions, which has led to a preference for Eulerian models, as they can cover the entire domain.

Over the years, several strategies have been proposed to address this limitation. A critical factor in improving Lagrangian model accuracy is the careful consideration of particle seeding on the surface from which they are tracked. In many cases, particles are seeded at the center of computational cells, which are often not uniformly distributed, especially near the walls where boundary layers are present. This distribution pattern significantly impacts the quality of Lagrangian predictions and studies suggest that a more uniform distribution of particles should be prioritized [58]. Another important approach involves performing a sensitivity analysis on the number of pathlines used [36].

Some methods have also been developed to account for the varying velocities of particles within the flow domain, as well as the tendency of red blood cells to accumulate in regions of higher velocity. Nikfar et al. tackled this by assigning a number of RBCs to each pathline based on its initial velocity and then multiplying the hemolysis index along each pathline by the number of RBCs assigned to it before calculating the overall hemolysis index for the device [56]. A similar method was used by Bornoff et al., where the initial velocities served as a weight in the overall hemolysis index computation [51].

As previously mentioned, the Eulerian approach has become the most widely adopted method for hemolysis prediction in recent years. Originally proposed by Garon and Farinas, it can be implemented using two formulations: a transport equation and a volume integral. Both methods are derived from the Lagrangian formulation $\mathrm{HI}3$ (Eq. 5) and are based on the concept of linearized blood damage [59]. The first is expressed in Eq. 8:

8 $$\left( {\frac{\partial }{\partial t} + {\boldsymbol{u}} \cdot \nabla } \right)HI^{\prime} = \left( {C\tau^{B} } \right)^{1/A}$$

In this transport equation, ${\boldsymbol{u}}$ is the velocity vector and ${\mathrm{HI}}{^{\prime}}$ represents the linearized hemolysis index. The total hemolysis index is obtained by evaluating ${\mathrm{HI}}^{\prime}$ at the device outlet and raising it to the power of $A.$

 The second method proposed is a simplification of the transport equation. It offers faster implementation and lower computational cost and can be introduced during post-processing. However, it is only valid under steady-state conditions where time dependence is negligible. This approach calculates an average hemolysis index, $\overline{\mathrm{HI}}$, using a volume integral of the linearized damage. The formulation also incorporates the flow rate *Q*, as shown in Eq. 9

9 $$\overline{\mathrm{HI}} = \left[ {\frac{1}{Q}*\mathop \smallint \limits_{V} \left( {C\tau^{{\mathrm{B}}} } \right)^{{1/{\mathrm{A}}}} dV} \right]^{{\mathrm{A}}}$$

Both Eulerian approaches can provide localized and global assessments of hemolysis risk, enabling meaningful comparisons with experimental data. They also address the primary limitation of Lagrangian models, namely the incomplete coverage of the fluid domain.

Despite their widespread use, these Eulerian models have two key limitations, as pointed out by Faghih and Sharp [60, 61]. First, they do not account for the spatial dependence of exposure time, limiting their validity to steady-state conditions or flows where velocity remains constant along streamlines. Second, there is a mathematical inaccuracy in distributing the exponent $A$ across the integral in the derivation of both formulations. As a result, these expressions are only mathematically valid when blood damage is uniformly distributed across the entire flow domain or the exponent $A$ has a unitary value, which is not the case in power-law models for hemolysis.

## Stress-Based Hemolysis Models

The choice of which stress definition to use in the power-law hemolysis model has been a longstanding topic of discussion. The model requires a scalar quantity to represent the viscous stress ${\boldsymbol{\upsigma}}$, which is a symmetric tensor defined in three dimensions (Eq. 10). This tensor is derived from the velocity vector ${\boldsymbol{u}}$ and is related to the strain rate tensor ${\boldsymbol{S}}$ through the dynamic viscosity $\mu$ of the fluid. It includes both normal stress components (${\sigma }_{ii}$) on the diagonal and shear components on the off-diagonal entries (${\sigma }_{ij}$).

10 $${\boldsymbol{\sigma}} = 2\mu {\boldsymbol{S}} = \mu \left( {\nabla {\boldsymbol{u}} + \nabla {\boldsymbol{u}}^{ \top } } \right) = \mu \left( {\begin{array}{*{20}c} {2\frac{\partial u}{{\partial x}}} & {\frac{\partial v}{{\partial x}} + \frac{\partial u}{{\partial y}}} & {\frac{\partial w}{{\partial x}} + \frac{\partial u}{{\partial z}}} \\ {^{\prime\prime}} & {2\frac{\partial v}{{\partial y}}} & {\frac{\partial w}{{\partial y}} + \frac{\partial v}{{\partial z}}} \\ {^{\prime\prime}} & {^{\prime\prime}} & {2\frac{\partial w}{{\partial z}}} \\ \end{array} } \right) = \left( {\begin{array}{*{20}c} {\sigma_{xx} } & {\sigma_{xy} } & {\sigma_{xz} } \\ {\sigma_{yx} } & {\sigma_{yy} } & {\sigma_{yz} } \\ {\sigma_{zx} } & {\sigma_{zy} } & {\sigma_{zz} } \\ \end{array} } \right)$$

### Von Mises-Like Equivalent Shear Stress

In literature, several methods employ the second invariant of the viscous stress tensor to reduce it to a resultant scalar stress ($\overline{\tau }$) suitable for the power-law formulation [49]. Among these, two approaches are predominantly used, both originating from the equation introduced by Bludszuweit [50]. This formulation adapts the concept of the Von Mises stress, traditionally applied in solid mechanics, to fluid-induced hemolysis modeling.

However, an error in Bludszuweit’s original derivation led to an expression that is mathematically inconsistent with the power-law model, a discrepancy highlighted by Faghih and Sharp [62]. Despite this, the original formulation for the resultant scalar stress ${\overline{\tau }}_{Bl}$, shown in Eq. 11, remains widely used in many studies [34, 52, 63–70].

11 $$\overline{\tau }_{Bl} = \left\{ {\frac{2}{3}\left[ {\sigma_{xx}^{2} + \sigma_{yy}^{2} + \sigma_{zz}^{2} } \right] - \frac{2}{3}\left[ {\sigma_{xx} \sigma_{yy} + \sigma_{yy} \sigma_{zz} + \sigma_{xx} \sigma_{zz} } \right] + 2\left[ {\sigma_{xy}^{2} + \sigma_{yz}^{2} + \sigma_{xz}^{2} } \right]} \right\}^{1/2}$$

The inconsistency becomes evident under pure shear conditions ($\tau ={\sigma }_{xy}$), where the equation yields ${\overline{\tau }}_{Bl}=\sqrt{2}{\sigma }_{xy}$, deviating from the conditions under which the power-law was originally formulated.

To address this, a corrected version of the scalar stress formulation has been established and is now commonly adopted. This version of the resultant scalar stress ${\overline{\tau }}_{eq}$ is presented in Eq. 12 and aligns accurately with the theoretical assumptions of the power-law model.

12 $$\overline{\tau }_{eq} = \left\{ {\frac{1}{3}\left[ {\sigma_{xx}^{2} + \sigma_{yy}^{2} + \sigma_{zz}^{2} } \right] - \frac{1}{3}\left[ {\sigma_{xx} \sigma_{yy} + \sigma_{yy} \sigma_{zz} + \sigma_{xx} \sigma_{zz} } \right] + \left[ {\sigma_{xy}^{2} + \sigma_{yz}^{2} + \sigma_{xz}^{2} } \right]} \right\}^{1/2}$$

### Shear and Extensional Stress

Both formulations for the equivalent shear stress discussed above aim to replicate the stress conditions underlying the original power-law model. However, it is important to note that the experiments at the foundation of this model were conducted under pure shear flow and do not consider extensional flow, which frequently occurs in blood-contacting devices. As demonstrated by Khoo et al., in rotary blood pumps, significant volumes of blood are subjected to extensional stress [71]. This occurs when different parts of a red blood cell experience different velocities, causing the cell to deform without the rotational motion typical of pure shear flow [5]. A classic example is found at sharp constrictions, where abrupt changes in cross-sectional area cause spatial acceleration of the fluid.

The distinct effects of pure extensional and shear flows on RBCs are evident in their mechanical responses. In extensional flow, the cell aligns its major axis with the principal strain axis, while in shear flow, a tank-treading motion takes place [71]. In the extensional case, only part of the membrane is subjected to higher stresses and the overall membrane tension remains constant [72]. In contrast, shear flow causes membrane tension to vary periodically and the rotating motion of the membrane leads to alternating exposure of different regions to peak stress, ultimately making it less damaging [72].

While RBC deformability under extensional flow has been investigated since 1992, significant comparative analyses of shear and extensional stresses only began to emerge around 2011 [73, 74]. That year, Down et al. published a CFD study based on Keshaviah’s earlier experimental work with capillary tubes [75]. Their numerical simulations helped characterize the flow regime near the capillary entrance, where Keshaviah had found that sharp contractions produce a greater hemolytic effect than tapered ones. Simulation results revealed that both shear and extensional stresses peak near the contraction corner, while remaining negligible upstream. Instead, downstream in the capillary, the numerical findings showed that only shear stress persists, whereas extensional stress drops off almost entirely.

These findings, taken together with Keshaviah’s experimental observations, indicate that hemolysis cannot be attributed solely to shear stress. If that were the case, the geometry of the entrance region would not play such a critical role.

Building on these insights, Yen et al. conducted both in silico and in vitro studies comparing sharp and tapered contractions, reaching conclusions closely aligned with those of Down et al. [76]. Their results further highlighted a strong correlation between hemolysis and extensional stress and suggested that traditional equivalent shear stress formulations inadequately capture the damaging effects of extensional flow.

Faghih and Sharp experimentally demonstrated that RBCs are significantly more sensitive to extensional stress than to shear stress [77]. Their results showed that shear stress must be approximately 34 times greater than extensional stress to produce an equivalent deformation. This relationship was used to derive an empirical weighting factor ${C}_{n}$, equal to $\frac{33.74}{\sqrt{3}}$, which modifies the traditional scalar stress formulation to emphasize the contribution of extensional stresses. The resulting stress ${\overline{\tau }}_{ext}$ is provided in Eq. 13:

13 $$\overline{\tau }_{ext} = \left\{ {C_{n}^{2} \left[ {\sigma_{xx}^{2} + \sigma_{yy}^{2} + \sigma_{zz}^{2} - \left( {\sigma_{xx} \sigma_{yy} + \sigma_{yy} \sigma_{zz} + \sigma_{xx} \sigma_{zz} } \right)} \right] + \left( {\sigma_{xy}^{2} + \sigma_{yz}^{2} + \sigma_{xz}^{2} } \right)} \right\}^{1/2}$$

This new formulation seeks to more accurately reflect the different cellular responses to extensional versus shear stress by assigning greater weight to the former. Supporting its validity, Maghouli et al. demonstrated that this new stress formulation outperforms that of Eq. 12 when applied to the Food and Drug Administration’s nozzle benchmark test case [78].

### Inclusion of Turbulence

In addition to incorporating extensional stresses in the power-law equation, substantial efforts have also been directed toward accounting for the effects of turbulence on red blood cells and the associated risk of hemolysis.

Turbulent flow is characterized by a viscous stress tensor and by fluctuating velocities, from which Reynolds stresses can be derived [79]. The latter do not represent physical forces but arise from the time-averaging of the Navier-Stokes equations and are often interpreted as momentum fluxes due to turbulent fluctuations. These stresses govern momentum transfer at macroscopic scales, where turbulence is evident, but at microscopic scales, comparable to RBC size, laminar and turbulent flows may not differ significantly, and the roles of Reynolds stresses and turbulent structures are still to be determined [80, 81].

In turbulent flows, the total stress tensor ${{\boldsymbol{\sigma}}}_{{\boldsymbol{t}}}$ can be expressed as the sum of the viscous stress tensor ${\boldsymbol{\sigma}}$ and the Reynolds stress tensor ${\boldsymbol{\sigma}}\boldsymbol{^{\prime}}$, shown in Eq. 14 [5]:

14 $${\boldsymbol{\sigma}}{^{\prime}} = - \rho \left( {\begin{array}{*{20}c} {\overline{{u^{{\prime}{2}} }} } & {\overline{u^{\prime}v^{\prime}} } & {\overline{u^{\prime}w^{\prime}} } \\ {{\prime\prime}} & {\overline{{v^{{\prime}{2}} }} } & {\overline{v^{\prime}w^{\prime}} } \\ {{\prime\prime}} & {{\prime\prime}} & {\overline{{z^{{\prime}{2}} }} } \\ \end{array} } \right)$$

where, $\rho$ is the fluid density and $u{\prime}$, $v{\prime}$, and $w{\prime}$ are fluctuating velocities whose mean products are derived from the averaging of the Navier-Stokes equations.

The influence of turbulence on blood damage has been a matter of debate since at least 1965 and despite decades of research, its exact contribution to RBC damage remains unresolved, making it one of the central open questions in hemolysis modeling [82]. Sutera proposed that turbulence could be disregarded when the time-averaged shear stress remains below the hemolytic threshold [83]. Yet, a definitive threshold for hemolysis in turbulent conditions has never been universally defined and it has not been conclusively demonstrated that turbulent fluctuations are harmless when the mean shear stress is low.

Research into turbulence-induced hemolysis began in the late 1960s, focusing primarily on turbulent jet flows [79, 84–89]. These studies yielded a wide range of hemolytic thresholds, from 400 Pa with an exposure time of 10^−5^ s to 5000 Pa with an exposure time of 10^−6^ s, underscoring the complexity and variability of the phenomenon [5].

Kameneva et al. proposed a different approach to study blood damage in turbulent conditions, questioning the reliability of turbulent jet studies in isolating the role of turbulence [90]. They introduced a flow loop system consisting of a centrifugal pump, a compliance chamber, and a glass capillary tube with a diameter of 1 mm and a length of 70 mm. They tested two suspensions of washed bovine RBCs with different viscosities across Reynolds numbers ranging from 300 to 5000. The different viscosities allowed to achieve either laminar or turbulent flow under identical macroscopic conditions (wall shear stress, pressure variation, and exposure time). Their results demonstrated an exponential increase in hemolysis with increasing Reynolds number, though the underlying mechanisms remain ambiguous.

Several hypotheses have emerged in the literature to explain this increase in RBC damage under turbulent conditions. Among the most commonly discussed are:

- Kolmogorov-scale eddies: one perspective suggests that only vortices with length scales comparable to RBC dimensions can inflict damage [79]. These small eddies are thought to cause localized stretching of the erythrocyte membrane [90]. However, direct measurement of turbulent structures at the RBC scale has not been achieved, leaving the validity of this mechanism unconfirmed [80]. Furthermore, the feasibility of eddies cascading down to cell-sized scales is debated, particularly given the dense, non-dilute nature of blood [81]. While some studies suggest a weak correlation between eddy scale and damage potential, it remains unclear whether Kolmogorov-scale eddies are truly the most destructive [80].
- Fatigue fracture: another hypothesis proposes that cyclic stretching of RBC membranes under turbulent flow may induce fatigue-related rupture [90]. Though only recently investigated, experimental evidence shows that continuous deformation compromises the deformability of RBCs [91, 92]. However, no specific studies have yet been carried out on the induction of cyclic load on RBCs by turbulent flows.
- Cell–cell interactions: Antiga and Steinman emphasized the role of interactions between adjacent RBCs, particularly due to the narrow intercellular spacing in dense suspensions [81]. In larger eddies or bulk flow, cells tend to move cohesively; however, in smaller eddies, local velocity gradients may produce different velocities between nearby cells. This relative motion could lead to shear stresses between cells, particularly within or between vortices. Antiga and Steinman estimated the magnitude of these stresses and highlighted the importance of including such interactions in models of turbulent blood flow.

The multifaceted nature of these mechanisms makes it difficult to isolate the true cause of turbulence-induced hemolysis. The current state of knowledge does not support the selection of a single dominant hypothesis, nor does it conclusively rule any out. Targeted experimental studies with refined setups are still required to resolve these uncertainties and finally establish the role of turbulence in blood damage.

Numerous attempts have been made to define flow features that could effectively account for turbulence within hemolysis models, particularly for integration into power-law formulations. Among these, Reynolds stresses have been a primary focus since at least 1984 [86].

An equivalent shear stress that incorporates both viscous and turbulent contributions can be defined substituting the viscous terms in Eq. 12 with total stress quantities [5]. However, this substitution is rarely implemented in blood damage modeling, where only viscous contributions are typically considered due to the non-physical meaning of Reynolds stresses and to the still unclear effect of turbulence on hemolysis.

Bludszuweit was among the first to include both Reynolds shear stresses and viscous shear stresses in hemolysis modeling [93]. Later studies expanded on this approach [64, 94, 95]; however, in 2016, Goubergrits et al. introduced a novel method to account for turbulence effects and showed the shortcomings of Reynolds shear stresses [96]. Instead of conventional time-averaged pathlines, they used instantaneous pathlines derived through procedural noise functions to generate divergence-free random vector fields. These vector fields matched the root mean square of the velocity fluctuations that occur in turbulent flows.

The comparison between their method and the use of unscaled Reynolds shear stresses revealed that they, respectively, provide hemolysis predictions three and sixty times higher than those based solely on viscous shear stresses. They also found that using a 0.05 scaling factor for the Reynolds shear stresses leads to blood damage predictions with the same magnitude but with different spatial distribution with respect to their new approach and to viscous shear stress predictions. Since viscous shear stress originates from physical mechanisms, Goubergrits et al. ultimately cautioned against the use of Reynolds shear stresses for blood damage modeling. Nevertheless, their approach has yet to gain traction in literature.

At present, Reynolds stresses are generally viewed as inadequate standalone indicators of hemolysis, largely due to their inability to reflect energy distributions across turbulent scales. Their relevance at the cellular level remains uncertain and the precise mechanical loading experienced by RBCs under turbulent conditions still needs to be further investigated [80]. Consequently, recent research has sought alternative variables to replace the viscous equivalent shear stress in power-law models of hemolysis.

## Energy Dissipation-Based Hemolysis Models

Since 1969, a correlation between blood damage and energy dissipation has been suggested by Bluestein et al. [97]. Later studies further developed the hypothesis that hemolysis is related to the viscous dissipation of turbulent kinetic energy. These works also aimed to explain the correlation observed for Reynolds shear stresses by associating it with the production of turbulent kinetic energy [98].

In 2014, Yen et al. conducted an experimental study to evaluate Reynolds and turbulent viscous shear stresses, starting from the idea that RBCs in turbulent flow are likely to be damaged only by vortices whose spatial scale is comparable to the size of the cells, while Reynolds shear stresses refer to a much larger scale [79]. Turbulent viscous shear stresses represent a physical force that arises from the interaction between fluid viscosity and the velocity gradients characterizing small-scale vortices. Yen et al. found that the turbulent viscous shear stresses hemolytic threshold is about one order of magnitude lower than that for Reynolds shear stresses.

The use of turbulent dissipation rate as a hemolysis predictor has also been introduced in computational modeling. In 2019, Wu et al. proposed a scalar representation of the shear stress for the power-law model based on turbulent dissipation rate and compared the predictive performance of their effective shear stress with the equivalent shear stress typically used in stress-based hemolysis models [99].

Wu et al. defined a total turbulent dissipation rate (${\upvarepsilon }_{tot}$) as in Eq. 15, which led to the effective shear stress (${\overline{\tau }}_{Wu}$) described by Eq. 16:

15 $$\varepsilon_{tot} = 2\upsilon {\boldsymbol{S}}_{{{\boldsymbol{ij}}}} {\boldsymbol{S}}_{{{\boldsymbol{ij}}}} = \varepsilon_{vis} + \varepsilon_{turb} = 2\upsilon \overline{{S_{ij} S_{ij} }} + 2\upsilon \overline{{S_{ij}{\prime} S_{ij}{\prime} }}$$

16 $$\overline{\tau }_{{{\mathrm{Wu}}}} = \mu \sqrt {2S_{ij} S_{ij} } = \sqrt {\mu \rho \varepsilon_{{{\mathrm{tot}}}} }$$

In these equations, $\upsilon$ is the kinematic viscosity, ${\varepsilon }_{vis}$ and ${\varepsilon }_{turb}$ are, respectively, the viscous and the turbulent dissipation rates and ${S}_{ij}{\prime}$ is the fluctuating rate of strain. Both ${\varepsilon }_{vis}$ and ${\varepsilon }_{turb}$ are treated as time-averaged quantities. Wu et al. conducted CFD simulations on three test cases from literature and found improved predictive accuracy of the power-law model when using the effective shear stress instead of the equivalent shear stress.

Torner et al. also investigated the relationship between equivalent shear stress and total dissipation rate [35]. They extended the definition of total turbulent dissipation rate by including the contribution of the modeled turbulent dissipation rate (${\varepsilon }_{mod}$), which arises from fluctuating turbulent flow that is not directly resolved in CFD simulations, unless performed using direct numerical simulations (DNS). This approach ensures that Torner’s effective shear stress (${\overline{\tau }}_{To}$) accounts for contributions from the mean flow as well as both the resolved and modeled turbulent flow components (Eq. 17).

17 $$\overline{\tau }_{{{\mathrm{To}}}} = \sqrt {\mu \rho \left( {\varepsilon_{{{\mathrm{vis}}}} + \varepsilon_{{{\mathrm{turb}}}} + \varepsilon_{\bmod } } \right)}$$

The comparison between effective shear stress values calculated with and without the turbulent dissipation components showed that the influence of turbulent shear stresses diminishes as stress magnitude increases. The most significant effects were observed at stress levels below 50 Pa. In Torner’s study, which focused on an axial blood pump, the viscous stress emerged as the dominant contributor to effective stress, while the turbulent stresses have a reduced impact on hemolysis. Nevertheless, turbulent stresses might still play a role in different flow conditions and devices and should not be neglected when developing a numerical hemolysis model. Moreover, their strong influence at lower stress levels makes them particularly relevant for predicting other forms of blood damage, such as von Willebrand factor cleavage and platelet activation. These phenomena occur at shear stress thresholds much lower than those that lead to red blood cell rupture [100].

Tobin and Manning also adopted an energy dissipation approach, developing a framework based on intermittency-corrected turbulent viscous shear stress (ICTVSS) [101]. Using large eddy simulations (LES), they implemented a numerical approach that accounts for the intermittent nature of dissipation in turbulent flows. In their model, the total dissipation rate includes both a viscous component and a turbulent component, derived from sub-grid scale stresses introduced by the filtering operation inherent in LES. However, an inconsistency arises in their definition of ${\varepsilon }_{tot}$ due to the use of dynamic viscosity $\mu$ instead of the kinematic viscosity $\upsilon$.

In 2018, Faghih and Sharp assessed the suitability of energy dissipation rate as a hemolysis predictor by comparing RBC membrane tension under various flow conditions characterized by the same energy dissipation rate [102]. They examined five cases: two laminar flows and three turbulent shear flows. Their findings revealed substantial differences in membrane tensions across the five conditions, suggesting that energy dissipation rate is not a universally reliable parameter for predicting hemolysis.

## Strain-Based Hemolysis Models

The stress-based approach is derived from a purely empirical correlation between flow and blood damage and it does not rely on mechanical or physical characteristics specific to red blood cells. These aspects represent shortcomings of this method and several studies have aimed to address them by deepening the investigation of the relationship between flow features and deformation of the red blood cell membrane. This has led to the development of strain-based models.

### Membrane Models

A first step in this direction was taken with the definition of a mechanical model for the RBC membrane proposed by Rand in 1964 [103]. This viscoelastic model fits Rand’s experimental curve describing the mechanical strain and breakdown of the membrane. It is composed of three elements: an elastic part, a viscous part, and a Kelvin body, which consists of an elastic and a viscous element arranged in parallel. This last component was inspired by the work of Katchalsky et al. on red blood cell membrane behavior during hemolysis [104].

Rand’s viscoelastic model was later adopted by other researchers to develop their strain-based models. In 1974, Richardson described the red blood cell as a flexible shell membrane and studied its deformation and hemolysis under uniform shear flow conditions [105]. For low strain rates, the membrane was modeled as a Hookean elastic material and the membrane displacements were computed. The maximum stresses found were then applied to Rand’s model to investigate the membrane behavior under high shear rates. This led to the definition of an equation for the prediction of strain, which includes a linear term, a periodic term, and a term that decreases exponentially over time. The linear term indicates the time required for hemolysis to occur.

Starting from Rand’s work, Chen et al. also developed a constitutive model that links fluid stresses to hemolysis [106]. First, they related the stresses to the tension in the RBC membrane. Then, they developed a viscoelastic model of the membrane from Rand’s formulation and employed the calculated cell membrane tension to predict the strain of the RBC membrane (${S}_{m}$). Experimental data were used to calibrate the model and a threshold value of strain was defined to indicate complete cell rupture (${S}_{c}$). The strain-based hemolysis model developed by Chen et al. is described in Online Appendix C. Chen et al. compared their strain-based model with the traditional power-law model, using two different sets of parameters. Only their model correctly predicted the increase in hemolysis between the different geometries tested, while the stress-based models showed the opposite trend.

In 2013, Arwatz et al. suggested a new model that also originates from Rand’s viscoelastic formulation [107]. They introduced two time constants: one to represent the rapid change in membrane permeability during RBC deformation and another to describe the progressive stiffening of the membrane over time. This model was fitted to experimental measurements collected using a Couette viscometer. The model was found to accurately reproduce hemolysis data for both short and long exposure times. However, it has not been applied to realistic cardiovascular devices.

### Droplet Models

In 2004, Arora et al. proposed a novel approach for hemolysis prediction based on RBC strain, which later served as the foundation for several other models [41]. In this model, the red blood cell is considered as a deformable liquid droplet, using a mathematical framework initially developed by Maffettone and Minale [108]. This model uses a morphology tensor to describe the red blood cell shape, which is approximated with an ellipsoid. The original formulation accounts for shape recovery when shear stress is reduced or removed and includes non-affine deformations of the ellipsoid. Arora added a third term to represent the tank-treading motion typical of red blood cells, which is absent in simple liquid droplets. This term ensures that the internal and external fluid velocities match the rotational velocity of the lipid bilayer surrounding the cell. This velocity is proportional to the flow shear rate. The model, described in Online Appendix D, also includes three parameters that describe the physical properties of red blood cells, based on experimental observations. Arora’s model is implemented in a Lagrangian framework, where red blood cell deformation is calculated at each step along the flow pathlines obtained from fluid flow simulations. Arora et al. used this strain-based model to assess hemolysis in a centrifugal pump, obtaining results that aligned well with experimental data [109].

In 2013, Pauli et al. implemented Arora’s strain-based hemolysis model using an Eulerian framework. They adopted a least-square finite element formulation to solve the convection-reaction system for tracking hemoglobin release within the flow field [110]. This method enabled the application of the strain-based model in unsteady simulations and large-scale devices. When comparing this approach with an Eulerian stress-based model, they found that the strain-based model better captured the reduction in exposure time that occurs in blood pumps as flow rate and rotational speed increase. Nevertheless, they also noted that further calibration was necessary to accurately reproduce experimental hemolysis values. An attempt to define new parameters for the power-law when used in a strain-based approach is made by Gesenhues et al., as discussed previously.

Recently, Dirkes et al. proposed a new Eulerian formulation of Arora’s model, after identifying that Pauli’s implementation had omitted parts of the original model that could be essential for accurate predictions [111]. Dirkes developed both a full-order Eulerian formulation and a simplified version that reduces the number of differential variables from six to only two. This simplification improves computational efficiency and enables the model to be applied to realistic devices more effectively. The simplified version is called tank-treading morphology (TTM) model and transforms the rotation equation that describes RBC motion into an algebraic equation, which is faster to solve. Both models proposed by Dirkes et al. are presented in Online Appendix D.

A comparison between full-order Eulerian, TTM, Pauli’s Eulerian and Arora’s original Lagrangian models showed that Pauli’s version fails to accurately reproduce Arora’s results, even under simple shear flow conditions. On the other hand, both Dirkes' models are able to correctly match Arora’s predictions. Additionally, the TTM model closely agrees with the full-order model in most simulation cases, while significantly reducing computational cost.

This model has also been employed to study hemolysis in three flow channels and, when compared to several stress-based models, the TTM model was the only one that qualitatively matched experimental results. This highlights its better suitability for capturing the effects of short and rapid peaks in shear stress [112].

To date, several extensions of Arora’s model have been proposed. Vitale et al. used Arora’s approach to compute red blood cell deformation and then included membrane poration and hemoglobin release [113]. Membrane poration is calculated based on changes in membrane energy, while hemoglobin release is defined as a mass transfer phenomenon through the pores, driven by the difference in hemoglobin concentration across the membrane.

Ezzeldin et al. replaced the ellipsoidal liquid droplet representation with a high-fidelity model of the red blood cell. They employed a coarse-grained molecular model that represents the spectrin cytoskeleton of the RBC [46]. In this model, the instantaneous deformation is determined from the coordinates of the nodes forming the RBC structure. These coordinates are used to compute the instantaneous moment of inertia and the eigenvalues of this tensor provide information on cell deformation. This model is a step toward a more accurate description of blood damage at the level of individual cells. However, further experimental data are still needed to improve hemolysis prediction and to develop a model based on the detection of pore formation on the membrane. In particular, the exact surface tension required to initiate pore formation is not known and depends on the loading rate.

Both models proposed by Vitale and Ezzeldin offer improved predictions of flow regions with shear rates below the hemolytic threshold when compared to Arora and traditional stress-based models.

Vitale’s model was used as a starting point by Poorkhalil et al. to develop an approach that combines two mechanisms: the permeation of hemoglobin through the membrane, which occurs before rupture and under low shear conditions, and the release of hemoglobin after the cell ruptures. Their model showed good accuracy when compared both to Couette tests and to data available in the literature [114].

### Cellular and Molecular Level Models

The inclusion of membrane poration in numerical hemolysis models has been the focus of several recent approaches. In 2017, Sohrabi and Liu implemented a model that links macroscopic flow conditions to nanoscale events such as membrane poration and hemoglobin release [47]. They developed a RBC membrane model based on spring connections and coupled it with lattice Boltzmann CFD simulations using an immersed boundary approach. This setup allowed them to determine strains and stresses acting on the RBC membrane. These results were then used to perform molecular dynamics simulations aimed at evaluating blood damage. Three submodels were included: one to assess whether the conditions for pore formation were met, one to estimate pore size and one to compute hemoglobin diffusion through the pores. Their model showed good agreement with the experimental studies conducted; however, due to its high computational cost, the method is difficult to fully apply to realistic devices. In fact, it was tested on only a few erythrocytes and then the overall hemolysis induced by the ventricular assist device under study was estimated through statistical methods.

Another multiscale approach combining CFD and molecular dynamics simulations was proposed in 2020 by Nikfar et al. [56]. This Lagrangian method uses shear stress values along sample pathlines to drive a coarse-grained RBC model that calculates membrane deformation. These deformations are then used to evaluate pore formation and size, followed by a diffusion equation to compute hemoglobin release through the pores. The model showed good agreement with experimental data obtained for two commercial pumps: the CentriMag® and the HeartMate II™. The first is a centrifugal magnetically levitated pump while the second is an axial pump with mechanical bearings.

In 2023, Xu et al. coupled CFD simulations with a transport dissipative particle dynamic model and a coarse-grained RBC damage model [115]. This method enables the evaluation of cell movement, deformation, and hemoglobin diffusion using the particle dynamic framework. However, the model has been applied to only four RBCs to represent the entire flow domain of an axial blood pump.

## Power-Law Constants

Most of the numerical hemolysis models presented in the previous sections require the definition of parameters for the power-law equation (presented in Eq. 2). The selection of these parameters has been a subject of discussion since the model was first introduced.

To date, more than a dozen parameter sets have been proposed in the literature. These sets show significant differences, mainly due to variations in the experimental setups, the blood donor species used, and the procedures for blood handling. Additionally, inaccuracies in the experimental tests contribute to this variability. The available sets can generally be divided into two main categories: parameters directly derived from Couette viscometer tests and parameters obtained through combination of experimental tests and numerical simulations on realistic devices.

The first category has been described in the section called “Experimental Foundations of Hemolysis Modeling.” The second category of parameter sets is summarized in Table 2 and is derived through a combination of experimental and numerical evaluations of hemolysis in realistic blood-contacting devices.

**Table 2:** Summary of parameter sets available in literature for the power-law ($\mathrm{HI}\left(\%\right)=C*{t}_{exp}^{A}*{\uptau }^{B}$), derived from combination of experiments and simulations

Source	C	A	B	Study Specifications
Fraser 2012 [116]	1.74500*10^−6^	0.77620	1.96300	Ovine; RBPs; Eu-T;${\overline{\tau }}_{Bl}$
Tobin 2020 [101]	2.70000*10^−7^	0.26400	2.50000	Bovine; FDA-N; Eu-T; ICTVSS
Mantegazza 2023 [117]	2.87500*10^−8^	0.50000	2.65000	Bovine; Capillary; Eu-T;${\overline{\tau }}_{eq}$
Torner 2023 [119]	1.00000*10^−7^	0.53920	1.82770	Bovine; FDA-N, FDA-P, Capillary; Eu-I,${\overline{\tau }}_{To}$
Torner 2023 [119]	4.25600*10^−22^	2.80730	10.28600	Bovine; FDA-N, FDA-P, Capillary; Eu-I,${\overline{\tau }}_{To}$
Blum 2025 [55]	3.51500*10^−5^	0.61400	1.79500	Bovine; FDA-P; Eu-I,$\overline{\tau }$
Sarfare 2025 [54]	1.27113*10^−4^	0.81460	1.61840	Bovine; CPAD; Eu-I,${\overline{\tau }}_{eq}$
Sarfare 2025 [54]	2.35330*10^−4^	0.83281	1.20098	Bovine; CPAD; Lag,${\overline{\tau }}_{eq}$

Under *Study Specifications*, the following abbreviations are used (together with those previously introduced in “Stress-based Hemolysis Models” and “Energy Dissipation-based Hemolysis Models” sections): *RBPs* Rotary blood pumps; *FDA-N* FDA nozzle benchmark, *FDA-P* FDA pump benchmark, *CPAD* cavopulmonary assist device, *Eu-T *Eulerian through transport equation, *Eu-I* Eulerian through integration, *Lag* Lagrangian

The first set in this category was derived by Fraser et al. in 2012 [116]. They used a gradient descent algorithm to fit the power-law parameters within an Eulerian formulation of the stress-based model, using hemolysis experiments and CFD simulations of commercial axial and centrifugal blood pumps. The resulting parameters demonstrated good performance in predicting hemolysis across different operating conditions for the same devices.

Tobin and Manning later employed the Food and Drug Administration (FDA) nozzle benchmark to calibrate the power-law parameters, starting from the set proposed by Ding et al. for bovine blood [101]. Their validation was based on experimental results by Jhun et al., who measured hemolysis in bovine blood under turbulent jet flow conditions [89]. While Tobin and Manning’s set captured the general hemolysis trend, it significantly overpredicted values at high Reynolds numbers. Their model implementation used an Eulerian framework and included intermittency-corrected turbulent viscous shear stresses.

The FDA nozzle was also employed to evaluate a set of parameters proposed by Mantegazza et al., based on the surrogate modeling framework developed by Craven et al. [117, 118]. This approach uses a Kriging surrogate model trained on CFD simulations to optimize the power-law parameters for a given device. However, the method is computationally expensive, requiring a large number of simulations and Craven’s original surrogate model was developed only for a glass capillary device using experimental data from Kameneva et al. [90]. This model was developed with the Eulerian formulation of the traditional power-law. Mantegazza’s results showed that, although the parameters defined by Craven yield good agreement with normalized hemolysis trends in the FDA nozzle, they cannot reproduce absolute hemolysis values accurately.

Craven’s CFD-based Kriging surrogate modeling approach was also recently used by Sarfare et al. to derive parameter sets for predicting the hemolysis risk associated with a cavopulmonary assist device for Fontan patients, developed as a von Kármán viscous impeller pump [54]. They applied this method to a specific geometry of the device and a single operating condition and determined two sets of parameters: one intended for an Eulerian implementation of the traditional power-law model and another for use in a Lagrangian framework. These parameter sets were then applied to predict hemolysis in an alternative geometry of the cavopulmonary assist device and were compared with experimental hemolysis measurements. The results showed that the derived parameters improve the accuracy of the predictions compared to the power-law sets obtained from Couette viscometer data. With the Eulerian approach, the discrepancies with respect to the experimental results are between 16 and 20%, while with the Lagrangian approach they were reduced to a range between 7 and 15%.

A more general approach was pursued by Torner et al. in 2023, who aimed to identify universal parameter sets applicable across multiple devices [119]. They used results from three well-documented hemolysis studies as reference and defined two statistical objective functions to guide a multi-objective particle swarm optimization algorithm. This led to the identification of two parameter sets: one constrained within literature-based bounds and one derived purely from mathematical optimization. Although the second set provided better agreement with experimental results, it might not be implementable in some CFD solvers due to floating-point exception errors that would render the simulation not solvable due to divisions by zero. The study used an Eulerian approach and incorporated total dissipation rate in the shear stress definition. Despite showing improvement over traditional parameter sets, the optimized ones could not achieve accurate predictions for all three benchmark cases.

Table 2 also reports a set of parameters recently proposed by Blum et al., which will be discussed in “Emerging Trends in Computational Hemolysis Prediction” section.

## Emerging Trends in Computational Hemolysis Prediction

The previous sections aimed to shed light on the broad field of numerical hemolysis models, presenting both outdated approaches and newer methodologies that are currently in use. In this section, the focus shifts to aspects that are still underexplored and to emerging trends in numerical hemolysis modeling.

### Effect of Hematocrit and Cell Interactions

Most numerical models found in the literature either do not explicitly model red blood cells or, if they do, use a one-way coupling with the flow field, without accounting for interactions between cells or between cells and the walls. However, RBCs represent a large volume fraction of blood and their interactions can influence both their mechanical behavior and the induced hemolysis [81]. Moreover, the effect of RBC presence on turbulence development remains under studied, as does the relationship between red blood cells and recirculation regions [81, 120].

Even hemolysis models developed at the cellular or molecular level often neglect cell–cell interaction. At present, most of these models simulate only a limited number of cells and do not account for their mutual interactions.

Some studies in literature have addressed the simulation of cell–cell interactions in blood flow [57, 121, 122]. These works use a variety of numerical approaches, which are beyond the scope of this review. However, most of them do not include any hemolysis evaluation.

Only recently, a few models have begun to include cell–cell interaction in hemolysis predictions. In 2023, Jędrzejczak et al. developed a model to assess hemolysis in atherosclerotic arteries using a population balance rheology model [123]. This approach allows RBCs to be described as either individual cells or agglomerated cells. They combined this with an Eulerian implementation of the power-law hemolysis model, modified to account only for non-agglomerated and non-hemolyzed cells. Their results showed a good correlation between vessel shape and hemolysis; however, this method has not yet been applied to blood-wetted devices and the hemolysis predictions have not been experimentally validated.

Another attempt to include RBC interactions in hemolysis modeling is presented in the PhD thesis of Valtchanov [124]. He developed CFD simulations using a fully Eulerian structural method to simulate the RBC membrane [125]. With this model, he studied the effect of RBC collisions under simplified conditions and for hematocrit levels up to 36%. His main finding was that including cell–cell interactions led to higher areal strain and increased hemoglobin diffusion through the membrane compared to simulations with single cells. However, this model is not yet applicable to realistic blood-wetted devices.

In 2023, Porcaro and Saeedipour employed resolved CFD-DEM (Discrete Element Method) simulations to evaluate hemolysis in microfluidic channels [48]. They used an immersed boundary approach to couple plasma with RBCs and modeled the cells as clusters of 10 overlapping spheres, connected by flexible bonds. This structure captures both cell deformability and membrane properties. The model also includes cell–cell interactions through two mechanisms: a soft-sphere approach to simulate physical contact between cells during flow and a Morse potential to represent RBC aggregation into rouleaux [126].

To compute hemolysis, they applied the strain-based method proposed by Ezzeldin et al. (see “Strain-based Hemolysis Models” section), which uses the inertia tensor calculated for each RBC. One of the main findings of their work concerns the choice of viscosity for the suspending fluid. They showed that using the viscosity of whole blood leads to an overprediction of hemolysis by about one order of magnitude. On the other hand, simulations using plasma viscosity resulted in more accurate predictions. Moreover, with plasma viscosity, hemolysis appears to be nearly independent from hematocrit, allowing for the use of lower values (around 10–15%) in simulations, which helps to reduce computational costs.

Nevertheless, these findings are not directly transferable to other applications and the model remains too computationally expensive to be used for realistic blood-wetted devices. To address this limitation, the research group is now working on an unresolved CFD-DEM approach. This method aims to upscale the model and simulate larger fluid domains with a more feasible computational cost [127].

An important aspect related to cell interactions that has not yet been included in numerical hemolysis modeling is the Fåhræus-Lindqvist effect. This phenomenon describes the decrease in the apparent viscosity of blood in small vessels (smaller than 300 μm), caused by the migration of red blood cells away from the vessel walls toward the center. This results in the formation of a near-wall layer which is free of red blood cells and has a lower viscosity, closer to that of plasma. This layer is known as the cell-free layer and it influences flow dynamics in the vessel, also leading to lower shear stresses and pressure losses [128–130].

A similar effect is believed to occur in the narrow gaps of ventricular assist devices, such as in the space between the rotor and volute [131]. However, this has not yet been included in CFD simulations or hemolysis predictions.

Knüppel et al. developed a viscosity model that can approximate the higher concentration of red blood cells in the center of small gaps, represented in their study by microchannels [132]. At present, the model has only been validated for blood with a hematocrit of 5%. Nonetheless, the authors aim to further develop the model for more realistic hematocrit values, in order to provide meaningful insights into shear stresses and pressure losses in these critical regions of ventricular assist devices. This would represent an important step toward improving hemolysis predictions for these devices.

### Inclusion of Turbulence

As discussed in “Stress-based Hemolysis Models” section, a common challenge in numerical hemolysis modeling is how to properly account for the influence of different flow conditions, especially turbulent ones, on hemolysis predictions. Among the various strategies developed over the years, energy dissipation and strain-based hemolysis models are currently the most widely used.

Nevertheless, these blood damage methods still rely on the traditional power-law formulation, which was originally developed under assumptions of constant shear rates and laminar flow. Some cellular- and molecular-level hemolysis models have been proposed to capture a broader range of RBC responses across different flow regimes; however, these are not yet applicable to complex or realistic device geometries.

Recently, Rydquist and Esmaily investigated the effect of turbulence on single RBCs using cell-level simulations in both laminar and turbulent conditions, while keeping the wall shear stress constant [133]. Their findings showed that turbulence induces significantly greater membrane deformation (up to 14% more) due to the presence of shear peaks. Interestingly, they also observed that increasing the turbulent scale does not necessarily lead to higher deformation. Despite these insights, a practical method for directly incorporating turbulent effects into numerical hemolysis prediction algorithms is still lacking.

In line with the work by Faghih and Sharp to modify the equivalent shear stress formulation to include extensional flows (see Eq. 13), Dirkes and Behr proposed an alternative formulation aimed at better capturing the impact of extensional and turbulent flow components [134]. They suggested including flow vorticity in hemolysis models, arguing that vorticity provides information on RBC deformation and alignment with flow direction. Their results showed that the stress experienced by RBCs tends to decrease as vorticity increases.

Equation 18 shows how the resultant scalar stress $\overline{\tau }$ can be modified to include the influence of vorticity:

18 $$\overline{\tau }_{Di} = \left( {1 + \delta \left( {\upsilon_{d} } \right)} \right)\overline{\tau }$$

In this formulation, $\delta \left({\upsilon }_{d}\right)$ is a dimensionless correction factor that represents the deviation between an effective stress (${\overline{\tau }}_{Di}$), which includes the influence of vorticity, and the traditional shear stress. The proposed form of $\delta$ is still preliminary and is based on the TTM model proposed by Dirkes starting from Arora’s work (see “Strain-based Hemolysis Models” section). The authors are currently conducting further studies using more realistic RBC models and targeted experiments to validate the approach.

### Machine Learning Techniques to Support Hemolysis Modeling

Machine learning techniques have not yet had a major impact on the field of numerical hemolysis modeling. The few available studies that use surrogate modeling mostly focus on identifying power-law parameters to improve the accuracy of hemolysis predictions.

Among the works discussed in “Power-Law Constants” section, Craven was the first to apply surrogate modeling to generate a new set of parameters aimed at better approximating hemolysis in specific devices [118]. His approach has been further explored in later studies [54, 117].

The set of parameters proposed by Blum et al. was also derived using probabilistic approaches and surrogate modeling techniques [55]. The goal of their study was to incorporate the variability inherent in experimental tests used to derive power-law parameters into numerical hemolysis predictions. Starting from the experimental data of Zhang et al., they used the Markov Chain Monte Carlo method to obtain stochastic distributions for the three parameters of the power-law model. The effect of different parameter samples on hemolysis predictions was evaluated using the FDA pump benchmark and a reduced-order model, which significantly reduced the computational time required for the study. This approach allowed for the propagation of experimental uncertainties into the hemolysis results, producing predictions that include not only a mean value but also associated uncertainty. This method helps improve the robustness and reliability of numerical hemolysis models; however, it still lacks the ability to accurately predict absolute values of hemolysis.

Overall, the use of surrogate modeling and machine learning is still quite new in the context of hemolysis prediction. Nonetheless, it offers strong potential to enhance the applicability of complex and computationally expensive models, especially those that simulate RBCs at the cellular level.

### Expansion of Experimental Data Sets

A clear evidence from this extensive review of numerical hemolysis prediction is the urgent need for more and better experimental data. This represents a big challenge, especially because the type of experiments that should be conducted depends strongly on the specific goal of the research.

The nozzle and pump FDA benchmarks were an important first step toward providing reference geometries and experimental datasets, and their impact has been significant. Both benchmarks have been widely used in the field of hemolysis prediction, despite the large uncertainties that characterize some of the available experimental results.

Since 2022, a working group formed during the annual *Blood Damage Workshop* has been developing a new benchmark aimed at evaluating different numerical hemolysis models using the same test case and providing reliable validation data.

The new benchmark is based on a plane channel flow tested under different flow conditions, ranging from laminar to turbulent regimes. To date, eight international laboratories are contributing to this effort, with some focusing on experimental testing and others on numerical simulations. The progress of this benchmark was presented at the *ESAO Congress* in 2025 and the first experimental data and results are expected to be available in 2026 [135].

The results of this new benchmark are expected to provide new, high-quality data on both flow characteristics and blood damage, which researchers can use to improve and validate existing numerical hemolysis models or as a foundation for developing new ones.

## Conclusion

In conclusion, since the earliest studies on hemolysis and the development of the first numerical prediction model, extensive work has been carried out to achieve accurate and reliable predictions that can support the design of cardiovascular devices. Today, numerical models are routinely used to improve the development of such devices. However, a universal and easily applicable model that can predict absolute hemolysis levels across different devices and flow conditions is still missing.

Future research should aim to create a robust and generalized modeling framework that provides consistent predictions regardless of the specific device geometry or simulation details. Several promising directions are emerging, including strain-based models and turbulence-aware approaches. These hold the potential to improve prediction accuracy, especially in complex or turbulent flow regimes.

At the same time, computational cost remains a significant barrier; particularly for high-fidelity models that simulate RBCs at the cellular level. Addressing this limitation is therefore essential to enable the application of advanced hemolysis models in realistic device simulations.

From a numerical standpoint, reducing computational cost requires progress on two complementary fronts: the acceleration of CFD simulations and the simplification of hemolysis modeling itself. On the CFD side, one promising strategy is the use of graphics processing units (GPUs) to accelerate flow solvers. Although the integration of GPUs into CFD frameworks remains technically challenging and is still an active area of research, several studies have demonstrated substantial reductions in computational time, albeit often at the expense of increased memory requirements [136]. On the modeling side, machine learning represents a particularly powerful tool for reducing the cost of hemolysis predictions. By shifting most of the computational burden to a training phase, approaches based on machine learning can enable the efficient use of complex hemolysis models that would otherwise be prohibitively expensive. This is especially relevant for cellular and molecular hemolysis models. A representative example is the work by Porcaro and Saeedipour, who developed an unresolved CFD-DEM approach in which drag and lift processes acting on RBCs are learned from a RBC reduced-order model and a resolved CFD-DEM approach based on immersed boundary simulations [127]. This strategy enables simulations of channel flow involving up to several hundred thousand RBCs, an achievement that would have been unobtainable with the fully resolved approach. Although they have not yet addressed hemolysis modeling, this approach suggests a potential pathway toward rethinking hemolysis prediction and enabling the wider adoption of sophisticated methods, including those simulating the RBC at the cellular or molecular level.

Beyond cost reduction, machine learning also opens new opportunities for adopting more mechanistic alternatives to the power-law formulation, which are inherently more complex and computationally demanding.

In this context, a promising direction for future research is the development of alternative hemolysis models that go beyond the widely used power-law approach. The power-law formulation is intrinsically empirical and, in many cases, too simplistic to serve as a universal model for hemolysis. As highlighted by Faghih and Sharp and by Mantegazza et al., new efforts should focus on models that are directly derived from the mechanisms underlying RBC damage [5, 117]. One possible strategy is to relate local flow-induced stresses to the mechanical tension in the RBC membrane and to develop predictive models for hemoglobin release. This approach is conceptually similar to cell–cell and molecular-level hemolysis models, but with the goal of capturing these effects without requiring full-scale microscale simulations. In this context, Faghih and Sharp identified a set of independent variables related to membrane tension, which could serve as a foundation for alternatives to the traditional power-law parameters of representative shear stress and exposure time [72]. Further exploration of these mechanistically informed variables may provide a path toward more accurate and generalizable hemolysis models.

In line with this mechanistic perspective, recent work by Krisher et al. proposed shear rate as a more suitable variable than shear stress for predicting hemolysis [137]. Unlike shear stress, shear rate is independent from fluid viscosity, and they found that it correlates well with blood damage over a wide range of hematocrit levels and medium viscosities. In contrast, when shear stress is considered, this correlation is lost and hemolysis exhibits a strong dependence on both blood parameters. These findings are particularly relevant in the context of developing more robust hemolysis models, and future studies should investigate shear rate-based formulations and assess their applicability across different flow conditions and experimental setups.

As repeatedly emphasized, there is also a strong need for more comprehensive and reliable experimental datasets. These are critical to validate numerical models and improve the understanding of hemolysis under diverse flow regimes and geometries. In this context, community-driven efforts such as the new benchmark initiated by the *Blood Damage Workshop* represent a fundamental step toward standardized validation and cross-model comparison.

Overall, while substantial progress has been made, continued collaboration and innovation are still needed to move closer to predictive, reliable, and widely applicable numerical hemolysis models.

## Supplementary Information

Below is the link to the electronic supplementary material.

Supplementary file1 (DOCX 74 kb)

## Data Availability

All data supporting the findings of this study are available within the paper.
