# Supplementary material for: From Early Models to Emerging Trends: The Evolution of Computational Hemolysis Prediction
Source: Ann Biomed Eng. 2026 Mar 19;54(7):2187–208. doi: 10.1007/s10439-026-04063-3 (PMC13291019; doi:10.1007/s10439-026-04063-3)
Supplement: Supplementary file 1 — Supplementary file1 (DOCX 74 kb) [file 10439_2026_4063_MOESM1_ESM.docx]

## **Supplementary Materials**

**Title:** From Early Models to Emerging Trends: The Evolution of Computational Hemolysis Prediction

**Journal:** Annals of Biomedical Engineering

**DOI:** 10.1007/s10439-026-04063-3

**Authors:**

1. Ilaria Guidetti, Politecnico di Milano, Department of Chemistry, Materials and Chemical Engineering “Giulio Natta”, LaBS, Piazza Leonardo da Vinci, 32 - 20133 Milano, Italy
   ORCID ID: 0009-0004-5890-2514
2. Maria Laura Costantino, Politecnico di Milano, Department of Chemistry, Materials and Chemical Engineering “Giulio Natta”, LaBS, Piazza Leonardo da Vinci, 32 - 20133 Milano, Italy
   ORCID ID: 0000-0001-6974-2692
3. Francesco De Gaetano, Politecnico di Milano, Department of Chemistry, Materials and Chemical Engineering “Giulio Natta”, LaBS, Piazza Leonardo da Vinci, 32 - 20133 Milano, Italy
   ORCID ID: 0000-0002-7597-020X

**Corresponding author:** Ilaria Guidetti - E-mail: [ilaria.guidetti@polimi.it](mailto:ilaria.guidetti@polimi.it)

## **Appendix A**

Despite the widespread use of Couette viscometers to evaluate blood damage and derive power-law parameters they are not the only devices that have been used for this purpose.

In 2022, Mei et al. proposed a blood-shearing device that uses a glass capillary and a syringe pump [1]. Using this setup, they derived the following parameters: $C$ = 8.329*10^-5^, $A$ = 0.581 and $B$ = 1.242.

Another parameter set derived from a capillary-based setup was proposed by Sivek et al., who developed a test bench comprising a reservoir, a centrifugal pump and a capillary tube [2]. Their results yielded the following parameters: $C$ = 4.82*10^-5^, $A$ = 0.514 and $B$ = 1.748.

These two sets are not reported in Table 1 due to their limited adoption in literature and the presence of potential sources of error in their experimental setups. Specifically, both methods use capillaries that are two to five times wider than the gaps in Couette viscometers, which raises concerns about the validity of assuming uniform shear stress equal to the maximum shear value throughout the blood volume. If this approximation is invalid, it could lead to overestimation of average shear stress and inaccuracies in the derived parameters. Furthermore, Sivek *et* al.'s setup includes a centrifugal pump whose contribution to blood damage is not accounted for in the parameters derivation, further compromising the reliability of the results.

**Appendix B**

Grigioni et al. demonstrated that $HI1$ does not accurately replicate the hemolysis index predicted by the power-law equation under constant shear stress [3]. In the same study, they advised against $HI2$, as it fails to distinguish between different RBC loading histories. In contrast, the other three formulations account for cumulative blood damage sustained by red blood cells over time.

$HI3$ employs the linear damage model introduced by Garon and Farinas, which is obtained raising the power-law equation to the exponent $1/A$ [4]. This transformation captures the nonlinear time dependency of hemolysis. $HI3$ integrates the linear damage along the pathline and then computes the hemolysis index by raising the result to the exponent $A$.

The formulation proposed by Grigioni et al., $HI4$, addresses the shortcomings of $HI1$ and $HI2$ by introducing the concept of a mechanical damage dose [3]. This dose accumulates over the pathline, increasing with time. At each time step, the total dose experienced in previous steps is computed and added to the damage that occurs at the current step.

$HI5$, developed by Goubergrits and Affeld, introduces the concept of an effective time step computed at each iteration to account for shear stress variations [5]. This effective time represents the exposure duration required to achieve the previous hemolysis index under the new shear stress condition. It is summed with the current time step to determine the subsequent hemolysis index.

Despite their different formulations, $HI3$ and $HI5$ have been found to yield almost identical results [6]. However, this equivalence has not been explicitly demonstrated yet. The following reasoning provides formal proof of their similarity. Equation B.1 reports the expanded form of $HI3$, in which $\tau\left( t_{i} \right)$ is written as $\tau_{i}$ to improve readability:

$HI3\left( \% \right)=C*\left( \Delta t_{1}*\tau_{1}^{B/A}+\Delta t_{2}*\tau_{2}^{B/A}+\ldots+\Delta t_{N}*\tau_{N}^{B/A} \right)^{A}$ (B.1)

Considering the same pathline, Equation 7 can be written as Equation B.2:

$HI5\left( \% \right)=HI5\left( \% \right)\left( t_{N} \right)=C*\left( \left( \frac{HI5\left( \% \right)\left( t_{N-1} \right)}{C*\tau_{N}^{B}} \right)^{1/A}+\Delta t_{N} \right)^{A}*\tau_{N}^{B}$ (B.2)

Equation B.3 can be derived transporting the term $\tau_{N}^{B}$ inside the brackets and simplifying:

$HI5\left( \% \right)\left( t_{N} \right)=C*\left( C^{A}*\left( HI5\left( \% \right)\left( t_{N-1} \right) \right)^{1/A}+\Delta t_{N}*\tau_{N}^{B/A} \right)^{A}$ (B.3)

The formulation obtained in Equation B.3 holds true for all the previous time steps; hence, we can rewrite it as in Equation B.4:

$HI5\left( \% \right)\left( t_{N} \right)=C*\left( C^{A}*\left( C*\left( C^{A}*\left( HI5\left( \% \right)\left( t_{N-2} \right) \right)^{1/A}+\Delta t_{N-1}*\tau_{N-1}^{B/A} \right)^{A} \right)^{1/A}+\Delta t_{N}*\tau_{N}^{B/A} \right)^{A}=C*\left( C^{A}*\left( HI5\left( \% \right)\left( t_{N-2} \right) \right)^{1/A}+\Delta t_{N-1}*\tau_{N-1}^{B/A}+\Delta t_{N}*\tau_{N}^{B/A} \right)^{A}$ (B.4)

Proceeding in the same manner until the first time step, Equation B.5 is obtained:

$HI5\left( \% \right)\left( t_{N} \right)=C*\left( C^{A}*\left( HI5\left( \% \right)\left( t_{0} \right) \right)^{1/A}+\Delta t_{1}*\tau_{1}^{B/A}+\Delta t_{2}*\tau_{2}^{B/A}+\ldots+\Delta t_{N-1}*\tau_{N-1}^{B/A}+\Delta t_{N}*\tau_{N}^{B/A} \right)^{A}$ (B.5)

Under the assumption that the blood damage was null at the beginning of the pathline, Equation B.6 can be written, which corresponds exactly to Equation B.1.

$HI5\left( \% \right)\left( t_{N} \right)=C*\left( \Delta t_{1}*\tau_{1}^{B/A}+\Delta t_{2}*\tau_{2}^{B/A}+\ldots+\Delta t_{N-1}*\tau_{N-1}^{B/A}+\Delta t_{N}*\tau_{N}^{B/A} \right)^{A}$ (B.6)

**Appendix C**

Equations C.1 to C.3 show the strain-based hemolysis model developed by Chen et al. to determine the index of hemolysis (${HI}_{Chen}$) within a Lagrangian framework [7]:

$\frac{S_{m}}{S_{c}}=\frac{4\alpha b}{5}\left( \bar{\tau}-\tau_{e} \right)\left[ C_{0}+C_{1}\left( 1-e^{-C_{2}t_{exp}} \right)+C_{3}t_{exp} \right]$ (C.1)

$F_{i}=\left\{ \begin{matrix} 1 & \left( \frac{S_{m}}{S_{c}} \right)_{i}>1 \\ 0 & otherwise \end{matrix} \right.$ (C.2)

${HI}_{Chen}=\frac{\sum_{i} F_{i}\left( \Delta Q \right)_{i}}{\sum_{i} \left( \Delta Q \right)_{i}}$ (C.3)

In these equations, $S_{m}$ is the strain of the RBC membrane, $S_{c}$ is the strain threshold value for complete cell rupture, $\tau_{e}$ is the stress required to deform the RBC into an ellipsoid without areal strain. The model requires six parameters ($\alpha$, $b$, $C_{0}$, $C_{1}$, $C_{2}$ and $C_{3}$), which were derived from experimental evaluations. The hemolysis index is then computed determining a rupture indicator $F_{i}$ for each streamline and performing a weighted average considering each streamline flow rate $\Delta Q$.

**Appendix D**

Arora's formulation is based on the calculation of the morphology tensor $\boldsymbol{S}_{\boldsymbol{M}}$ (Equation D.1), from which the instantaneous shape distortion $D$ of the red blood cell can be derived, as in Equation D.2, by evaluating the lengths of the ellipsoid's semi-axes ($L_{e}$ and $B_{e}$) [8]. This distortion is then used in Equation D.3 to define the effective shear stress $\bar{\tau}_{Ar}$, which can be used in the power-law model to predict the hemolysis rate.

$\frac{\partial\boldsymbol{S}_{\boldsymbol{M}}}{\partial t}+\boldsymbol{u}\cdot\nabla\boldsymbol{S}_{\boldsymbol{M}}-\left[ \boldsymbol{\Omega}\cdot\boldsymbol{S}_{\boldsymbol{M}}-\boldsymbol{S}_{\boldsymbol{M}}\cdot\boldsymbol{\Omega} \right]=-f_{1}\left[ \boldsymbol{S}_{\boldsymbol{M}}-g\left( \boldsymbol{S}_{\boldsymbol{M}} \right)\boldsymbol{I} \right]+f_{2}\left[ \tilde{\boldsymbol{E}}\cdot\boldsymbol{S}_{\boldsymbol{M}}+\boldsymbol{S}_{\boldsymbol{M}}\cdot\tilde{\boldsymbol{E}} \right]+f_{3}\left[ \tilde{\boldsymbol{W}}\cdot\boldsymbol{S}_{\boldsymbol{M}}-\boldsymbol{S}_{\boldsymbol{M}}\cdot\tilde{\boldsymbol{W}} \right]$ (D.1)

$D=\frac{L_{e}-B_{e}}{L_{e}+B_{e}}$ (D.2)

$\bar{\tau}_{Ar}=\mu\frac{2Df_{1}}{\left( 1-D^{2} \right)f_{2}}$ (D.3)

In these equations, $\boldsymbol{\Omega}$ is the rotation rate of the rotating frame, $\boldsymbol{I}$ is the identity matrix, $\tilde{\boldsymbol{E}}$ and $\tilde{\boldsymbol{W}}$ are the relative rate of strain and relative vorticity tensor, respectively. $f_{1}$, $f_{2}$ and $f_{3}$ are parameters derived from experiments and $g$ is a function that returns the ratio between the third and the second invariant of the variable, multiplied by three.

Equation D.4 shows the full-order Eulerian implementation of Arora’s model while Equation D.5 shows the simplified tank-treading morphology model, both proposed by Dirkes et al. [9]:

$\frac{\partial\boldsymbol{S}_{\boldsymbol{M}}}{\partial t}+\boldsymbol{u}\cdot\nabla\boldsymbol{S}_{\boldsymbol{M}}=-f_{1}\left[ \boldsymbol{S}_{\boldsymbol{M}}-g\left( \boldsymbol{S}_{\boldsymbol{M}} \right)\boldsymbol{I} \right]+f_{2}\left[ \hat{\boldsymbol{E}}\cdot\boldsymbol{S}_{\boldsymbol{M}}+\boldsymbol{S}_{\boldsymbol{M}}\cdot\hat{\boldsymbol{E}} \right]+\frac{f_{2}}{f_{3}}\left[ \boldsymbol{(}\boldsymbol{E}\boldsymbol{-}\hat{\boldsymbol{E}}\boldsymbol{)}\cdot\boldsymbol{S}_{\boldsymbol{M}}+\boldsymbol{S}_{\boldsymbol{M}}\cdot\boldsymbol{(}\boldsymbol{E}\boldsymbol{-}\hat{\boldsymbol{E}}\boldsymbol{)} \right]+\left[ \boldsymbol{W}\cdot\boldsymbol{S}_{\boldsymbol{M}}-\boldsymbol{S}_{\boldsymbol{M}}\cdot\boldsymbol{W} \right]$ (D.4)

$\frac{\partial\lambda_{i}}{\partial t}+\boldsymbol{u}\cdot\nabla\lambda_{i}=-f_{1}\left[ \lambda_{i}-g\left( \boldsymbol{\Lambda} \right) \right]+2f_{2}\lambda_{i}\tilde{E}_{ii}$ (D.5)

In these equations, $\boldsymbol{E}$ and $\boldsymbol{W}$ are the symmetric rate of strain and the antisymmetric vorticity tensor, respectively; while $\hat{\boldsymbol{E}}$ is a projection of the eigenvectors $\boldsymbol{Q}_{\boldsymbol{\lambda}}$ of $\boldsymbol{S}_{\boldsymbol{M}}$ and causes RBC deformation. On the contrary, $\boldsymbol{E-}\hat{\boldsymbol{E}}$ causes its rotation. $\lambda_{i}$ represents the eigenvalues of $\boldsymbol{S}_{\boldsymbol{M}}$, $\boldsymbol{\Lambda}$ is the diagonal tensor of $\lambda_{i}$ and $\tilde{E}_{ii}$ is the transformation of $\boldsymbol{E}$ to the eigenbasis of $\boldsymbol{S}_{\boldsymbol{M}}$. In this model, a differentiation between tank-treading and tumbling motion is included by imposing $\boldsymbol{Q}_{\boldsymbol{\lambda}}\boldsymbol{=0}$ in case of tumbling.

**References**

[1] X. Mei, B. Lu, P. Wu, and L. Zhang, “In vitro study of red blood cell and VWF damage in mechanical circulatory support devices based on blood-shearing platform,” *Proc. Inst. Mech. Eng. H*, vol. 236, no. 6, pp. 860–866, Jun. 2022, doi: 10.1177/09544119221088420.

[2] A. D. Sivek, “Experimental investigation of mechanical blood damage relevant to the operation of circulatory-assist devices,” PhD Thesis, University of Pittsburgh, 2015.

[3] M. Grigioni, U. Morbiducci, G. D’Avenio, G. Di Benedetto, and C. Del Gaudio, “A novel formulation for blood trauma prediction by a modified power-law mathematical model,” *Biomech. Model. Mechanobiol.*, vol. 4, no. 4, pp. 249–260, Dec. 2005, doi: 10.1007/s10237-005-0005-y.

[4] A. Garon and M.-I. Farinas, “Fast Three-dimensional Numerical Hemolysis Approximation,” *Artif. Organs*, vol. 28, no. 11, pp. 1016–1025, 2004, doi: https://doi.org/10.1111/j.1525-1594.2004.00026.x.

[5] L. Goubergrits and K. Affeld, “Numerical Estimation of Blood Damage in Artificial Organs,” *Artif. Organs*, vol. 28, no. 5, pp. 499–507, 2004, doi: https://doi.org/10.1111/j.1525-1594.2004.07265.x.

[6] M. E. Taskin, K. H. Fraser, T. Zhang, C. Wu, B. P. Griffith, and Z. J. Wu, “Evaluation of Eulerian and Lagrangian models for hemolysis estimation,” *ASAIO Journal*, vol. 58, no. 4, pp. 363–372, Jul. 2012, doi: 10.1097/MAT.0b013e318254833b.

[7] Y. Chen, T. L. Kent, and M. K. Sharp, “Testing of Models of Flow‐Induced Hemolysis in Blood Flow Through Hypodermic Needles,” *Artif. Organs*, vol. 37, no. 3, pp. 256–266, Mar. 2013, doi: 10.1111/j.1525-1594.2012.01569.x.

[8] D. Arora, M. Behr, and M. Pasquali, “A Tensor-Based Measure for Estimating Blood Damage,” *Artif. Organs*, vol. 28, pp. 1002–1015, 2004, doi: https://doi.org/10.1111/j.1525-1594.2004.00072.x.

[9] N. Dirkes, F. Key, and M. Behr, “Eulerian formulation of the tensor-based morphology equations for strain-based blood damage modeling,” *Comput. Methods Appl. Mech. Eng.*, vol. 426, Jun. 2024, doi: 10.1016/j.cma.2024.116979.
